# Enhanced exosome secretion regulated by microglial P2X7R in the medullary dorsal horn contributes to pulpitis-induced pain

**DOI:** 10.1186/s13578-025-01363-4

**Published:** 2025-02-22

**Authors:** Jing Zhang, Zhuo Yu, Mingjun Wang, Xiaoning Kang, Xiaoke Wu, Fengjiao Yang, Lu Yang, Shukai Sun, Li-an Wu

**Affiliations:** State Key Laboratory of Oral & Maxillofacial Reconstruction and Regeneration, National Clinical Research Center for Oral Diseases, Shaanxi Clinical Research Center for Oral Diseases, Department of Pediatric Dentistry, School of Stomatology, The Fourth Military Medical University, Xi’an, 710032 China

**Keywords:** Pulpitis, Microglia, P2X7R, Exosome, Pain

## Abstract

**Background:**

Pulpitis is a prevalent oral disease characterized by severe pain. The activation of microglia in the medullary dorsal horn (MDH) is reportedly essential for the central sensitization mechanism associated with pulpitis. The P2X7 receptor (P2X7R) on microglia can trigger the secretion of exosomes enriched with IL-1β, which is involved in inflammation. Thus, we hypothesized that the enhanced exosome secretion regulated by microglial P2X7R in the MDH contributes to pulpitis-induced pain.

**Methods:**

An experimental pulpitis model was established in male SD rats to observe pain behaviors. Immunofluorescence staining, western blotting and quantitative real-time PCR were used to analyze the expression of IL-1β and Rab27a, a key protein secreted by exosomes during nociceptive processes. The effects of the exosome inhibitor GW4869 and the P2X7R antagonist Brilliant Blue G (BBG) on microglial P2X7R, exosome secretion and inflammation in the pulpitis model were analyzed. In vitro, microglial cells were cultured to collect exosomes, and stimulation with lipopolysaccharide (LPS), oxidized ATP (oxATP) and GW4869 altered the secretion of exosomes containing IL-1β.

**Results:**

In the experimental pulpitis model, the microglial exosome secretion and inflammatory factor release in the MDH were both correlated with the extent of pulpitis-induced pain, with the highest expression occurring on the 7th day. GW4869 and BBG inhibited Rab27a and IL-1β expression, reducing pulpitis-induced pain. In addition, exosomes were successfully extracted by ultracentrifugation in vitro, wherein LPS treatment promoted exosome secretion but GW4869 had the opposite effects on the secretion of exosomes and the IL-1β. Moreover, P2X7R inhibition by oxATP diminished exosome secretion, leading to a reduction in inflammatory responses.

**Conclusion:**

This study highlights the regulatory role of microglial P2X7R in increased exosome secretion, indicating the potential utility of P2X7R as a promising target for pulpitis therapy. Our research highlights a new pulpitis mechanism in which exosomes enriched with IL-1β contribute to pulpitis-induced pain, suggesting the crucial roles of exosomes as pain biomarkers and harmful signaling molecules during pulpitis.

**Supplementary Information:**

The online version contains supplementary material available at 10.1186/s13578-025-01363-4.

## Background

Pulpitis is a prevalent dental condition primarily caused by severe dental caries that is characterized by orofacial pain[1]. Pulpitis may be reversible in its early stage; however, without timely intervention, it progresses to become irreversible[2]. Contemporary dental treatment employs a combination of local interventions and drug-assisted treatments to manage pulpitis development[3]. Unfortunately, patients in remote areas, especially during epidemic periods, may face challenges in receiving timely treatment, resulting in severe orofacial pain and dental anxiety[4]. Consequently, pulpitis-induced pain significantly impacts the patients’ oral health and quality of life[5]. However, the exact mechanism of pulpitis pain remains elusive, emphasizing its importance in the treatment of this condition[6, 7].

Pulpitis reportedly lowers the pain threshold of nerve fiber-containing endings[8]. The activation of pulpal nociceptors promotes central sensitization of the medullary dorsal horn (MDH)[9]. Microglial activation in the MDH has been reported to be essential for the central sensitization mechanism associated with pulpitis[10]. The P2X7 receptor (P2X7R), an ATP-gated cation channel, is enriched on the surface of microglia and plays a crucial role in the development of inflammation and pain[11–13]. Blocking P2X7R has been shown to decrease central sensitization in nociceptive neurons[14]. Our previous research demonstrated that microglial P2X7R in the MDH promoted the release of IL-1β and contributed to the occurrence of pulpitis-induced pain[15, 16]. Notably, previous studies have indicated that P2X7R activation could mediate the secretion of extracellular vesicles (EVs), especially exosomes and microvesicles containing inflammatory factors[17–21]. However, the relationship between P2X7R and exosomes in pulpitis-induced pain has not been investigated.

Exosomes, with a diameter of 40–100 nm, are classified as membrane vesicles. Exosomes serve as potential biomarkers in various diseases[22–25]. Notably, as signal transmission mediators, exosomes participate in both inflammatory and neuropathic pain[26, 27]. Interestingly, it has been suggested that brain-derived exosomes can regulate the pain threshold and hyperalgesia[28]. Furthermore, nociceptive signals mediated by microglia can be transmitted to neurons via exosomes[29]. Recently, studies have identified Rab27a as a key protein secreted by exosomes that controls the fusion of multivesicular endosomes to the plasma membrane, leading to exosome secretion[30]. Rab27a also plays a significant role in nociceptive processes[31, 32]. Despite these findings, the role of exosomes in pulpitis-induced pain remains unclear.

In this study, we explored the potential relationship between microglial P2X7R and exosomes in the pathogenesis and development of pulpitis-induced pain. We found that increased exosome secretion was regulated by microglial P2X7R in the MDH and contributed to pulpitis-induced pain. In addition, lipopolysaccharide (LPS)-stimulated exosomes were enriched with IL-1β, which is associated with the transmission of pain signals. Importantly, both inhibiting the secretion of exosomes and administering P2X7R antagonists alleviated pain behaviors and inflammatory responses. In summary, our findings confirm that exosomes play an important role in pulpitis-induced pain modulation and that microglial P2X7R regulates pulpitis-induced pain by controlling the secretion of inflammatory exosomes containing IL-1β.

## Materials and methods

### Animals and the establishment of an experimental pulpitis model

**Fig. 1:**
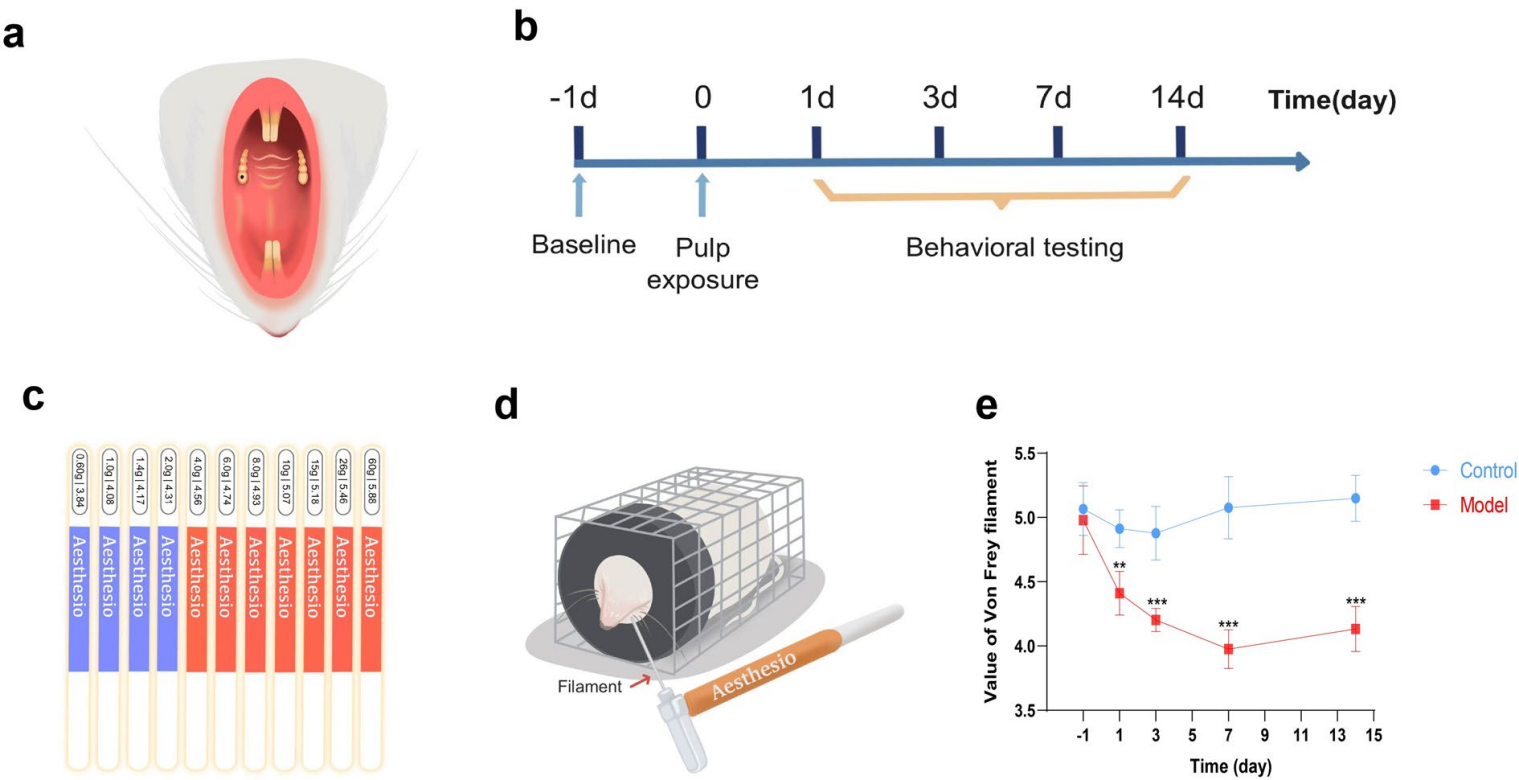
The establishment of experimental pulpitis model and pain thresholds of rats. **a** Exposure of dental pulp. **b** Experimental flow chart. **c** Size of von Frey filaments. **d** Diagram of behavioral testing. **e** Pain thresholds of rats (n = 6 rats in both groups). **P < 0.01, ***P < 0.001

The Animal Use and Care Committee of the Fourth Military Medical University approved all the animal experiments conducted in this study. All methods and procedures were performed in accordance with the National Institutes of Health Guidelines for the Care and Use of Laboratory Animals and with the consent of the ethical regulations for animal research. Male Sprague–Dawley rats (200–250 g) were obtained from the Animal Center of the Fourth Military Medical University and kept at a controlled temperature (22 ± 2 °C) on a 12 h’s light–dark cycle. To establish an experimental pulpitis model, we used the dental pulp exposure procedure described previously[15, 33–35]. All the rats were divided into 5 groups and 4 groups underwent the pulp exposure procedure according to the pulp exposure time (1d, 3d, 7d, and 14d), except the rats in the control group. Firstly, the rats were narcotized with pentobarbital (50 mg/kg) via intraperitoneal injection and fixed on an operating table facing up. The mouths of the rats were then opened using retractors. Pulp exposure was achieved on the maxillary left first molar using a rotating ball-shaped dental bur (Fig. 1a). Following the procedure, the rats were returned to their respective cages.

### Cell culture and cell stimulation

HMC3 cells purchased from ATCC were used as the microglial cell line. The cells were cultured in minimum Eagle's medium (MEM) supplemented with 10% fetal bovine serum (FBS; Gibco, United States) and 1% penicillin/streptomycin. The cells were cultured at 37 °C with 5% CO_2_. For treatment, 1 µg/ml LPS (Sigma‒Aldrich, United States) was added to the medium for 24 h of incubation. Furthermore, 5 µM GW4869 (MCE, China) or 30 µM oxidized ATP (oxATP; Sigma‒Aldrich, United States) was separately added to the appropriate groups 2 h prior to LPS treatment. RNA and protein were extracted 24 h after stimulation.

### Behavioral assessments

The facial mechanical pain threshold was assessed using von Frey filaments (Danmic Global, United States) at various time points during exposure (Fig. 1b). The von Frey filaments ranged in size from 3.84 to 5.88 (Fig. 1c). The methodology employed in this study followed the procedures outlined in previous studies[15, 16]. First, the rats were placed in a designated mesh cage (6 cm × 6 cm × 20 cm) for 30 min to acclimate to the testing environment, ensuring a calm and steady state with the rats oriented toward the front. Von Frey filaments were subsequently applied to the facial skin of each rat near the operated tooth, as illustrated in Fig. 1d. Starting with the filament with a size of 3.84, each filament was tested 10 times, with a minimum of a 5-s interval between consecutive tests. If positive facial responses, such as rapid withdrawal, biting, or multiple head shaking events, occurred more than 5 times, the rat's response was deemed effective, and the filament size was recorded. Otherwise, the rat was subjected to stimulation once again using the next larger filament after 3 min of rest.

### Stereotactic injection of GW4869 and intraperitoneal injection of Brilliant Blue G (BBG)

Before stereotactic surgery, 12 rats were randomly divided into two groups: the vehicle (pulpitis 7d + dimethyl sulfoxide (DMSO)) and GW4869 (pulpitis 7d + GW4869) groups. After 7 days of pulp exposure, GW4869 was injected as described previously[14]. Either 3 μl of GW4869 or an equivalent volume of 20% DMSO was injected into the MDH (lateral: 1.5–2.0 mm; posterior: 1.5–2.0 mm, referenced to the obex) at a rate of 0.2 μl/min. To confirm the accuracy of the site, fluorescent gold was injected into the MDH, and the area was observed using a VS 200 scanner (Olympus, Japan). Additionally, 18 rats were randomly divided into three groups: the Sham (saline), vehicle (pulpitis 7d + saline), and BBG (pulpitis 7d + BBG) groups. Saline or BBG (50 mg/kg) was administered intraperitoneally (i.p.) every other day for 7 days. The chosen dose was selected on the basis of previous studies[36, 37].

### Immunofluorescence staining

The remaining MDH tissue was sectioned into slices (25 μm) using a freezing microtome. These slices were subjected to three washes with phosphate-buffered saline (PBS) and subsequently blocked with 10% donkey serum for 1 h. Next, the sections were incubated with primary antibodies for 16–18 h at 4 °C. After an additional three washes, the sections were exposed to Alexa Fluor secondary antibodies for 3 h and counterstained with DAPI (1:1,000) for 10 min at room temperature. A comprehensive list of the relevant antibodies is provided in Table S1. The fluorescence signals were visualized using an FV3000 laser scanning confocal microscope (Olympus, Japan).

### Exosome isolation and identification

As shown in Fig. 4a, exosomes were isolated from microglial supernatants via differential centrifugation. To eliminate dead cells and debris, the supernatant was subjected to sequential centrifugation at 300 × g for 10 min, 2,000 × g for 10 min, and 10,000 × g for 30 min at 4 °C. Subsequently, ultracentrifugation at 100,000 × g for 70 min was performed, and the resulting pellet was washed with PBS before undergoing an additional centrifugation step at 100,000 × g for 70 min at 4 °C (Beckman Coulter). Finally, the exosomes were resuspended in 100 μl of PBS and stored at −80 °C. Transmission electron microscopy (TEM) was used to visualize the morphology of the exosomes, and a Flow Nano analyzer was used to assess their size and secretion volume. Western blot analysis was employed to identify exosome protein markers, including CD63 and CD81.

### Western blot

For western blot, the remaining MDH tissues and HMC3 cells were collected on ice and homogenized in radioimmunoprecipitation assay lysis buffer (Beyotime Biotechnology, China). The protein concentration was determined via a bicinchoninic acid assay. The proteins were separated via SDS‒PAGE (Bio-Rad, United States) and subsequently transferred to PVDF membranes (Millipore, United States). After the membranes were washed three times with TBS-T, they were blocked with 5% bovine serum albumin (BSA) for 2 h. Primary antibodies (P2X7R, Rab27a, IL-1β, CD63, CD81, and β-actin) were then added, and the samples were incubated overnight at 4 °C. Following three washes with TBS-T, the membranes were incubated with secondary antibodies (1:4,000) for 1 h. Detailed information about the antibodies is provided in Table S2. Finally, images of the protein bands were acquired using an automatic exposure instrument, and analysis was conducted using ImageJ software.

### Immunocytochemistry

HMC3 cells were fixed with 4% paraformaldehyde, washed with PBS and permeabilized with 0.3% Triton X-100 for 20 min. After the samples were blocked with 5% BSA for 30 min at room temperature, they were incubated with primary antibody (Rab27a, 1:1,000) overnight at 4 °C. Following three washes with PBS, the cells were stained with an Alexa Fluor 488-conjugated secondary antibody (1:500) for 2 h and then with DAPI for 5 min at room temperature. Each slide was then washed again, slightly dried, and sealed with an antifluorescence quenching sealant. Images were captured using an FV1000 microscope (Olympus, Japan).

### Quantitative real-time PCR (qRT–PCR)

Total RNA from whole-cell lysates and MDH tissues was extracted using TRIzol reagent (Thermo Fisher Scientific, United States), from which cDNA was synthesized using PrimeScript RT Master Mix (TaKaRa, Japan). The synthesized cDNA was employed for qPCR amplification with an ABI 7500 detection system (Applied Biosystems, United States). The primer sequences used are provided in Table S3.

### Statistical analysis

All data were expressed as mean ± standard deviation, and each experiment was independently repeated at least three times. GraphPad Prism 8.0 was used for statistical analysis of the experimental results. Student's t test was utilized to assess differences between two groups, while multiple group comparisons were conducted via one-way ANOVA with Tukey's test for post hoc analysis. Two-way ANOVA was used to analyze experiments involving two dependent variables. Pearson correlation analysis, which was performed using the Coloc 2 plug-in of ImageJ software, was employed for two-variable correlation analysis. A *p* value of < 0.05 was considered to indicate statistical significance.

## Results

### Mechanical hyperalgesia in a rat model of experimental pulpitis

In this study, we established an experimental pulpitis model by exposing the dental pulp of rats (Fig. 1a) and assessed changes in the pain threshold via a behavioral test in which the left facial cheeks of the rats were stimulated with von Frey filaments. The model diagram and experimental flow chart are presented in Fig. 1b, and the range of von Frey filaments and the behavioral test diagram are depicted in Fig. 1c–d. Our behavioral results revealed that the model group had a significantly lower pain threshold than that in the control group. With prolonged dental pulp exposure, the pain threshold gradually decreased, reaching its lowest value on day 7 (Fig. 1e). That is to say, the rat with pulp exposure felt most painful on day 7, and the degree of pain in pulpitis model rats was more obvious than that in normal rats in the whole 14 days. These findings suggest that the experimental pulpitis model was successfully established and that the changes in pulpitis-induced mechanical hyperalgesia were time dependent.

### The secretion of exosomes and IL-1β was enhanced in the MDH of pulpitis rats

**Fig. 2:**
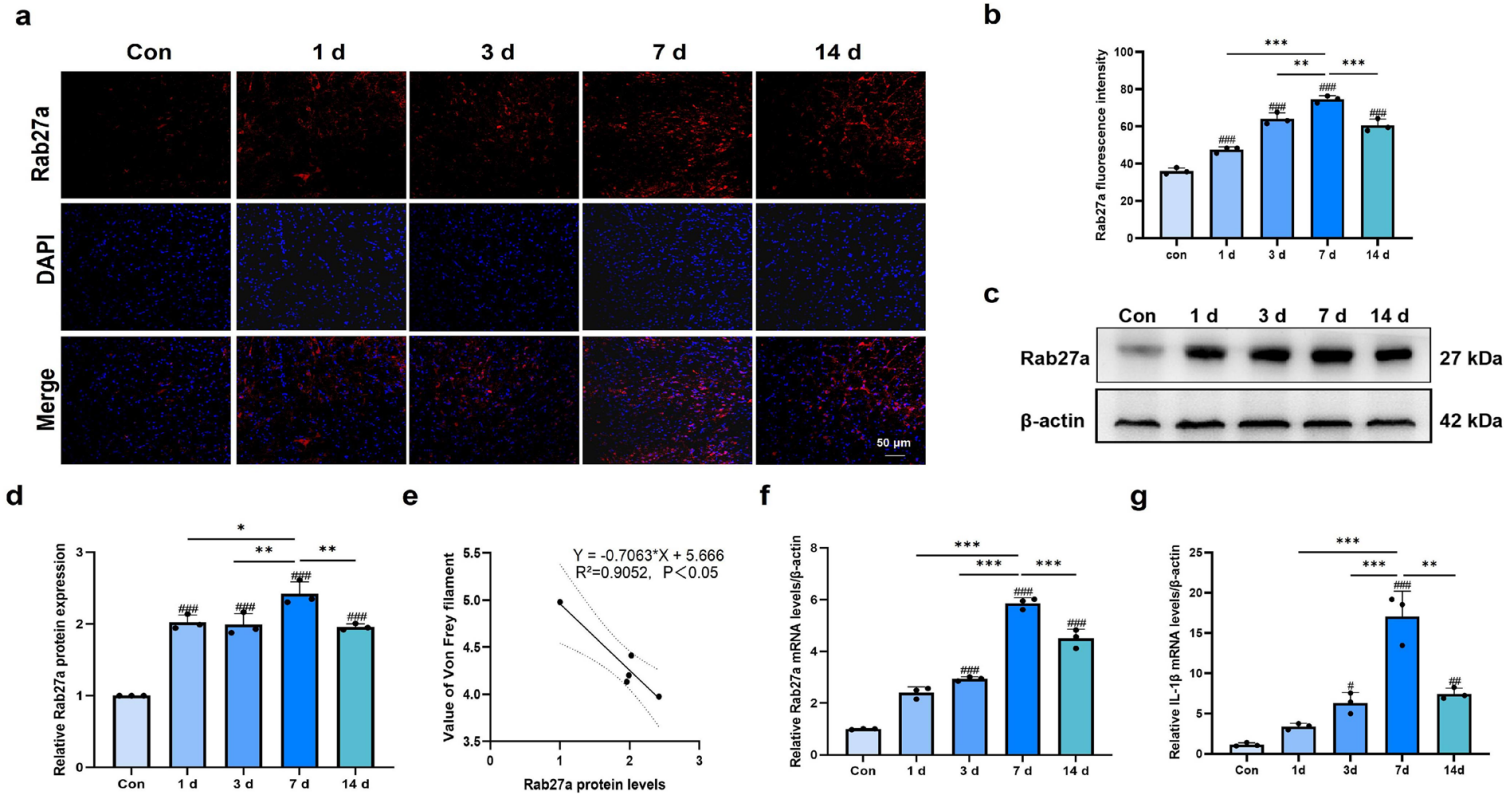
Rab27a was upregulated in MDH of pulpitis rats and negatively associated with pain thresholds (n = 3 rats for each experiment at each time point). **a–b** Immunofluorescence staining results of Rab27a in MDH (scale bar, 50 μm). **c–d** Rab27a expression in MDH of pulpitis rats was significantly increased than that in control rats. **e** Relationship between Rab27a expression and pain thresholds. **f** Rab27a and IL-1β mRNA levels in MDH were measured by qRT-PCR. *P < 0.05, **P < 0.01, ***P < 0.001; ^*#*^*P* < 0.05, ^*##*^*P* < 0.01, ^*###*^*P* < 0.001

Fresh MDH tissues were collected at different time points, and the expression of Rab27a, the selected representative protein secreted by exosomes, was evaluated. Immunofluorescence staining revealed a significant increase in Rab27a expression after pulp exposure, which was correlated with exposure time, as shown in Fig. 2a–b. Western blot analysis revealed that the protein levels of Rab27a changed over time, with the highest expression occurring on day 7 (Fig. 2c–d). Furthermore, we observed a negative correlation between increased Rab27a expression and the pain threshold (Fig. 2e). To substantiate these findings, qRT-PCR was used to assess the mRNA levels of Rab27a and the pain-related signaling molecule IL-1β. Compared with the control, pulpitis induction resulted in increases in the secretion of exosomes and the inflammatory factor IL-1β in the MDH, which exhibited similar trends (Fig. 2f–g). These results indicate that the expression of Rab27a and inflammatory factors increase after pulp exposure, demonstrating a positive correlation with the severity of pulpitis-induced pain. The increased secretion of exosomes correlated with exacerbated inflammation, which contributed to persistent pain.

### GW4869 alleviated pulpitis-induced pain and inhibited the inflammatory reaction

**Fig. 3:**
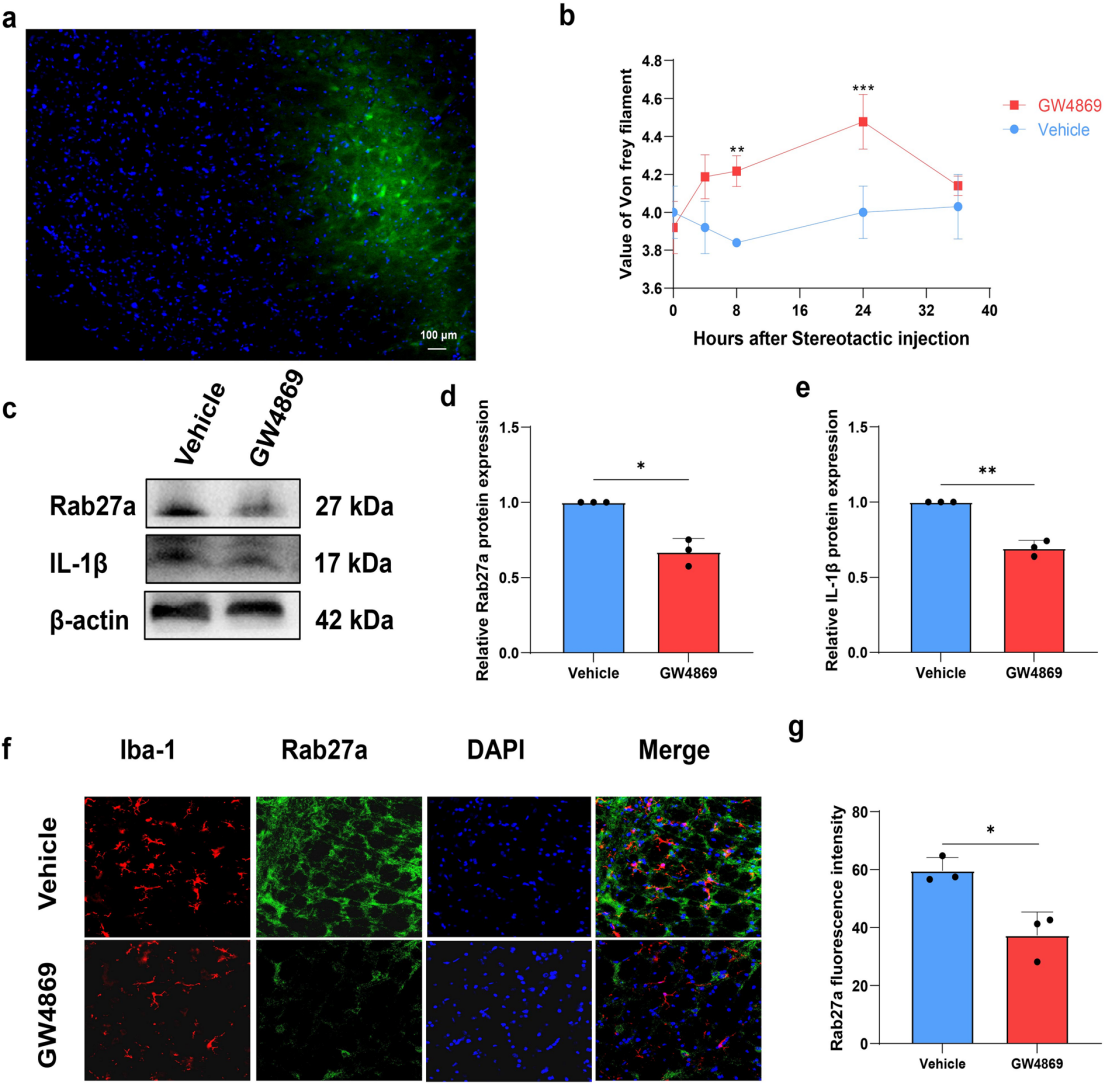
GW4869 alleviated pulpitis pain and inhibited the expression of Rab27a and IL-1β. **a** Site map of the MDH area.** b** Changes in pain thresholds after treatment with GW4869. **c–e** Rab27a and IL-1β expression in MDH were tested by western blot. **f–g** Rab27a in microglia was decreased in MDH after stereotactic injection of GW4869. The spot’s position is represented as a black dot (n = 3 rats in both groups). *P < 0.05, **P < 0.01

To determine the role of exosomes in pulpitis-induced pain modulation, we utilized the exosome inhibitor GW4869 in a stereotactic injection experiment. Fluorescent gold dye was first injected to confirm the accuracy of the site to ensure successful MDH targeting for subsequent experiments (Fig. 3a). Pulpitis rats that received stereotactic injection of DMSO were considered the vehicle group. Following GW4869 or DMSO injection, the pain thresholds of the rats were assessed. Notably, compared with vehicle, GW4869 increased the pain threshold and alleviated pain in pulpitis model rats, with the greatest increase in the pain threshold observed 24 h after GW4869 treatment (Fig. 3b). These findings suggest that inhibiting exosome secretion could block the transmission of pain signals and hinder inflammation reactions, thereby relieving pain. In addition, the expression levels of Rab27a and IL-1β were examined via western blotting. GW4869 downregulated both Rab27a and IL-1β, indicating a decrease in inflammatory reactions (Fig. 3c–e). Additionally, to determine the effect of GW4869 on microglial exosomes, double immunofluorescence staining was performed to evaluate the colocalization of Iba-1 and Rab27a, and significant inhibition of microglial Rab27a expression was observed after GW4869 treatment (Fig. 3f–g).

### The P2X7R antagonist BBG downregulated Rab27a and IL-1β in vivo

**Fig. 4:**
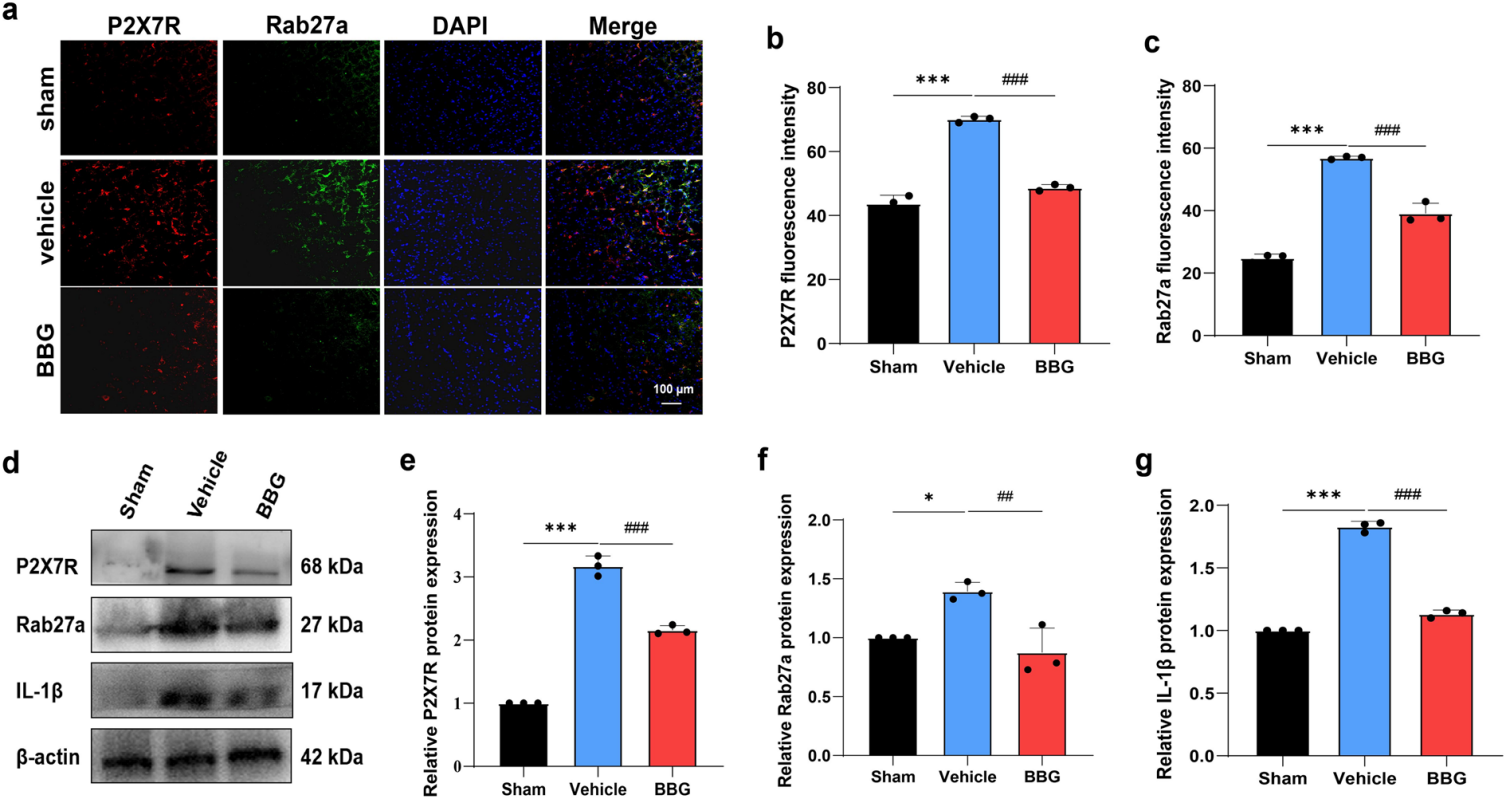
P2X7R antagonist BBG decreased Rab27a and IL-1β expression. **a–c** Immunofluorescence images and analysis of P2X7 (red) and Rab27a (green) (Scale bar, 100 μm). **d–g** BBG decreased P2X7, Rab27a and IL-1β expression. The spot’s position is represented as a black dot (n = 3 rats in each group). *P < 0.05, ***P < 0.001; ^##^*P* < 0.01, ^###^*P* < 0.001

Given that P2X7R predominantly exists in microglia in the central nervous system (CNS)[38], we conducted a P2X7R blockade experiment using the P2X7R antagonist BBG. Previous studies have suggested that P2X7R can control exosome secretion[39, 40]. Therefore, we aimed to investigate whether P2X7R regulates microglial Rab27a expression and IL-1β secretion in the experimental pulpitis model. As depicted in Fig. 4a–c, in vivo BBG administration reduced both P2X7R and Rab27a expression, indicating reduced exosome secretion. Moreover, the western blot results were consistent with the immunofluorescence staining results, as both P2X7R and Rab27a expression were inhibited after treatment with BBG (Fig. 4d–f). Importantly, the level of the representative proinflammatory cytokine IL-1β was also reduced after P2X7R blockade (Fig. 4g). These results suggest that changes in the secretion of exosomes and the inflammatory factor IL-1β are associated with P2X7R expression.

### GW4869 and oxATP suppressed microglial exosome secretion in vitro

**Fig. 5:**
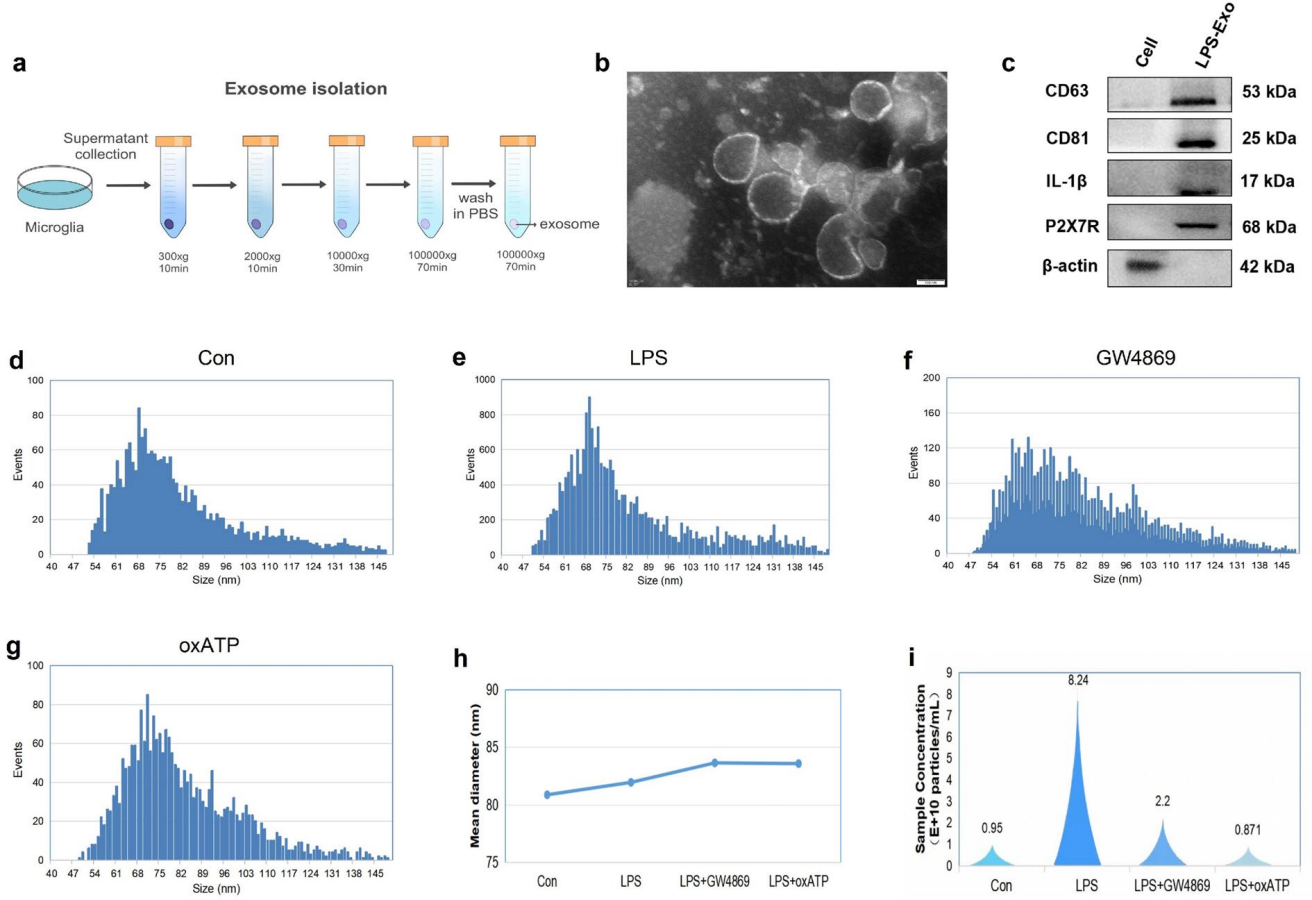
Characterization and secretion changes of microglia-derived exosomes. **a** Flow chart of exosome isolation. **b** Morphology image of exosomes acquired by TEM. Scale bar = 100 nm. **c** Western blot image showing the expression of exosome markers (CD63 and CD81) and inflammatory markers (IL-1β and P2X7) in LPS-stimulated exosomes. **d–i** Analysis of the particle number and size of exosomes from the different treatment groups by a Flow Nano Analyzer

To further confirm the role of P2X7R in exosome secretion in an inflammatory environment, we cultured microglial cells in vitro before extracting the exosomes via ultracentrifugation (Fig. 5a). TEM revealed that the exosomes exhibited a bilayer membrane structure and cup-shaped morphology (Fig. 5b). Following stimulation with 1 μM LPS, the western blot results revealed that exosome-specific proteins, including CD63 and CD81, were expressed. Intriguingly, IL-1β and P2X7R were also present in the LPS-stimulated exosomes (Fig. 5c), suggesting that the release of IL-1β from P2X7R-activated microglia is mediated primarily by exosomes, which is consistent with the findings of previous studies[41, 42]. We also detected the size of the obtained exosomes using nanoflow analysis[43–45]. And the results revealed that the diameters of the exosomes ranged from 40 to 160 nm, which aligns with the diameters of typical exosomes, and the averaged exosome size was comparable among the different groups (Fig. 5d–h). The concentration and particles of exosomes can be used to quantitatively evaluate exosome secretion[46]. Our findings indicated that, compared with the control, exposure to LPS increased microglial exosome secretion, whereas treatment with GW4869 and oxATP inhibited this secretion. Notably, the inhibitory effect of oxATP was particularly significant (Fig. 5i), highlighting that oxATP, a P2X7R antagonist, can be used as an effective suppressor of microglial exosome secretion.

### Inhibiting microglial exosome secretion decreased the release of the inflammatory factor IL-1β

**Fig. 6:**
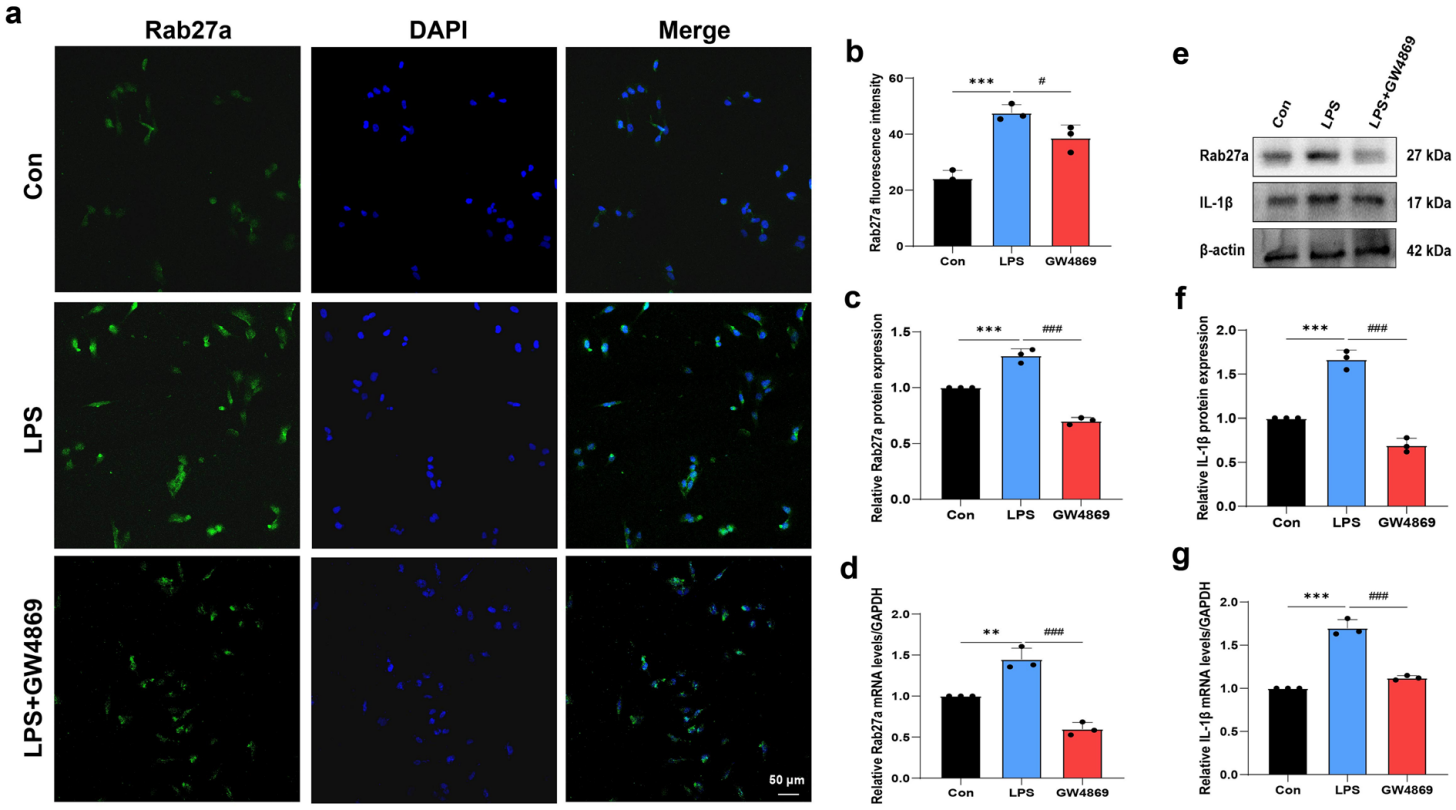
GW4869 inhibited the secretion of exosomes and inflammatory cytokine in vitro (n = 3 experiments per group). **a–b** Immunofluorescence images and analysis of Rab27a in different groups. **c–e** Western blot image showing the decreased expression of Rab27a and IL-1β treated with GW4869. **f–g** Rab27a and IL-1β mRNA levels in microglia were measured by qRT-PCR. **P < 0.01, ***P < 0.001; ^#^*P* < 0.05, ^###^*P* < 0.001

In the experimental pulpitis model, GW4869 administration led to pain relief and suppressed the expression of microglial Rab27a. These in vivo results suggest that treatment with GW4869 prevents the secretion of inflammatory exosomes from microglia, thus alleviating pain sensitivity. To validate this hypothesis in vitro, three distinct experimental techniques were employed. First, immunofluorescence staining revealed that LPS increased the expression level of Rab27a, but the exosome inhibitor GW4869 reversed this effect (Fig. 6a–b). We also observed that the protein and mRNA levels of Rab27a and IL-1β were markedly increased after LPS treatment, whereas GW4869 inhibited the LPS-induced upregulation of these factors (Fig. 6c–g), indicating that inhibiting exosome secretion negatively impacted the release of IL-1β in an inflammatory environment.

### LPS promoted the secretion of exosomes from microglia through P2X7R

**Fig. 7:**
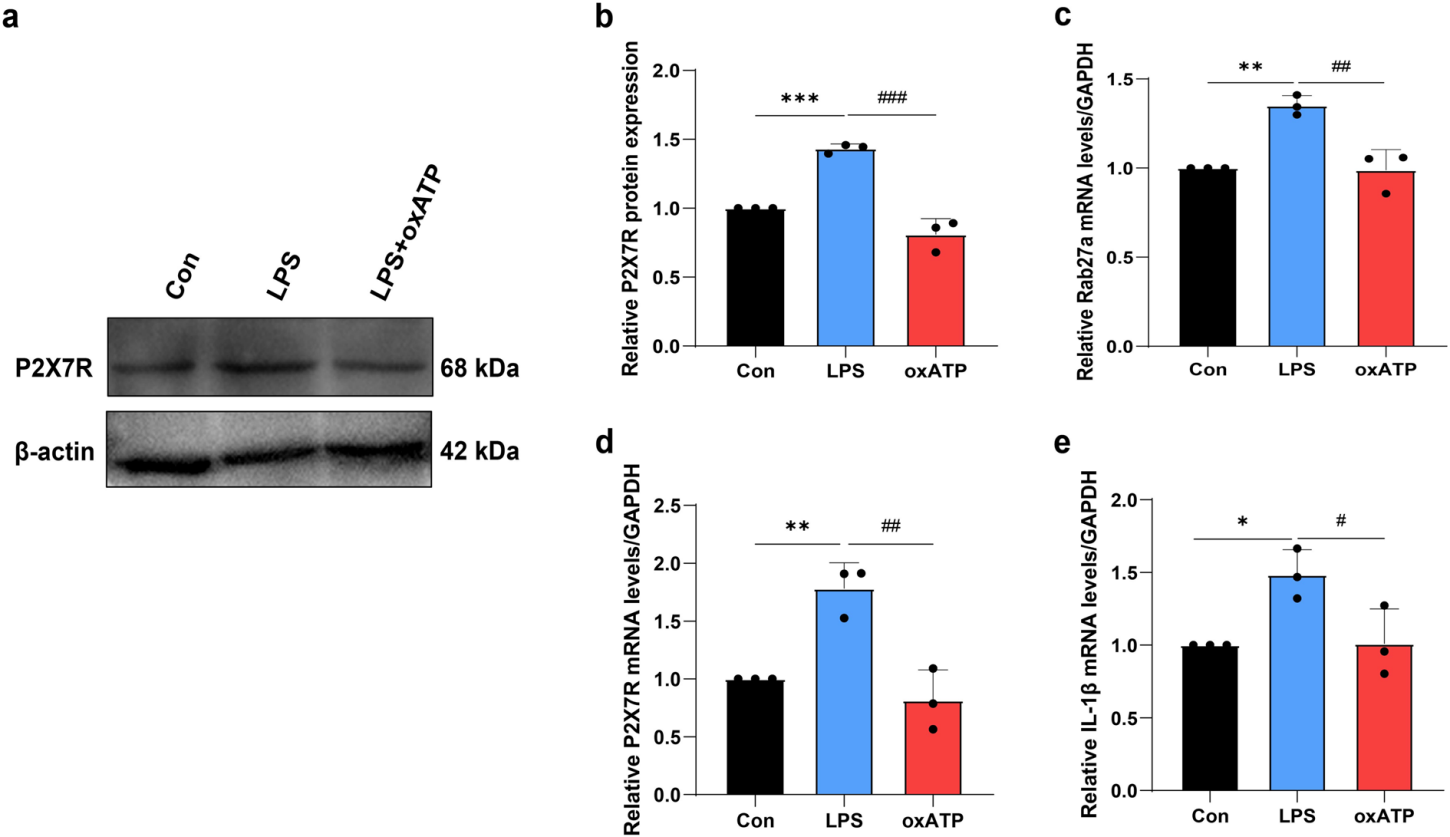
P2X7R antagonist oxATP decreased P2X7, Rab27a, and IL-1β expression in vitro (n = 3 experiments per group). **a–b** P2X7R was inhibited by using oxATP. **c–e** P2X7, Rab27a, and IL-1β mRNA levels were decreased with oxATP treatment. *P < 0.05, **P < 0.01, ***P < 0.001; ^#^*P* < 0.05, ^##^*P* < 0.01, ^###^*P* < 0.001

Microglia are activated upon LPS stimulation, which is accompanied by a marked increase in P2X7R expression[47–49]. However, treatment with the P2X7 antagonist oxATP effectively inhibited the upregulation of P2X7R (Fig. 7a–b). To confirm the relationship between P2X7R and microglial exosomes, oxATP was administered to LPS-stimulated microglia. The qRT-PCR results demonstrated that, compared with the control, LPS stimulation increased the mRNA levels of P2X7R, Rab27a, and IL-1β, whereas oxATP had the opposite effect on the secretion of Rab27a and IL-1β (Fig. 7c–e). These findings indicate that P2X7R activation plays a pivotal role in the inflammation-driven secretion of exosomes from microglia.

## Discussion

Pulpitis is the most prevalent dental pulp-related condition and contributes to approximately one‒sixth of orofacial pain cases each year worldwide[50]. The exacerbation of pain observed in irreversible pulpitis underscores the urgency of timely intervention[51, 52]. Hence, there is a crucial need to investigate new pain-related biomarkers and therapeutic targets for pulpitis. While the pain-inducing role of microglial P2X7R in the MDH in the experimental pulpitis model has been documented[14–16], the relevant molecular mechanisms warrant further exploration. In this study, employing molecular biology techniques and cell biology analyses, we propose that exosomes could serve as pivotal pulpitis-induced pain markers in the MDH and that IL-1β is released from these enhanced exosomes via microglial P2X7R regulation.

Initially, we established an experimental pulpitis model by exposing the dental pulp of rats, which has been widely used by many research teams. In particular, this animal model of pulpitis has been used by our research team for years with several publications[15, 53–55]. Pain thresholds at different time points were recorded after conducting the von Frey test. To ensure a free and stable experimental environment for the rats, we utilized a specifically designed porous metal mesh cage with a circular gap in the fore area, allowing the facial regions of the rats to protrude. A significant decrease in the pain threshold of pulpitis rats compared with that of control rats was observed. Stimulation of the pulpitis rats with von Frey filaments elicited head shaking, withdrawal, facial scratching, and even vocalizations, indicating the manifestation of pulpitis-induced pain. The peak of painful symptoms was observed on day 7 after pulp exposure, which is consistent with previous studies that validated the reliability of the von Frey test in evaluating orofacial pain behaviors[15, 56, 57].

While pulpitis pain is linked to microglial activation in the MDH[10, 58], the mechanisms by which microglia induce neuronal hyperexcitability remain unclear. Exosomes, which are crucial for intercellular signaling and act as biomarkers in various diseases, have been implicated in both inflammatory and neuropathic pain[59]. In the chronic constriction injury (CCI) model, brain-derived exosomes were shown to participate in pain signal transmission[28]. Rab27a, a protein excreted by exosomes, is involved in the processing of inflammation-induced pain[32]. Microglia-derived exosomes can carry proinflammatory cytokines and deliver them directly into recipient cells[19]. Additionally, the levels of proinflammatory cytokines within exosomes are strongly regulated by inflammatory stimuli such as LPS and ATP[60]. Building upon these insights, we explored how exosomes in the MDH are involved in the development of pulpitis. The expression levels of Rab27a and the inflammatory cytokine IL-1β were measured at different time points via western blotting, qRT‒PCR, and immunofluorescence staining. The expression of both Rab27a and IL-1β exhibited consistent trends, reaching their highest levels on day 7 after pulp exposure. Notably, Rab27a protein levels were inversely related to the pain threshold, preliminarily suggesting that exosomes and inflammatory factors are involved in the regulation of pulpitis-induced pain.

GW4869 is as a noncompetitive inhibitor of cell membrane neutral sphingomyelinase (nSMase), which catalyzes the production of ceramide by hydrolyzing membrane sphingolipids. Ceramide, in turn, plays a crucial role in endosomal sorting complexes required for transport machinery-independent exosome generation and secretion. Therefore, GW4869 significantly inhibits the secretion of exosomes[61]. Upon stereotactic injection of GW4869 into the rat MDH, we observed that pulpitis pain was alleviated, particularly 24 h later. The mechanism underlying this effect may be attributed to the GW4869-mediated reduction in the secretion of inflammatory exosomes enriched in IL-1β via Rab27a downregulation. This, in turn, inhibits the accumulation of inflammation and the aggravation of pain. Further investigations are warranted to explore the underlying mechanisms mediating exosome secretion in pulpitis-induced pain.

In our previous study, we confirmed that microglial P2X7R is involved in pulpitis-induced pain[16]. Notably, recent research has focused on the purinergic signaling pathway's regulation of the secretion and function of EVs. Specifically, P2X7R is recognized as an upstream regulator of exosome secretion[62–64]. P2X7R siRNA relieves pain behaviors by inhibiting exosome secretion from microglia, and P2X7R antagonists such as A804598 and FTY720 alleviate neuroinflammation caused by brain injury via exosome inhibition[65–67]. More importantly, the secretion of IL-1β via microglia-derived EVs played a key role in pain sensation due to P2X7R activation in the spinal nerve ligation (SNL) model[67]. However, the relationship between microglial P2X7R and exosome secretion has not been validated in a pulpitis pain model. BBG, a specific P2X7R antagonist that can penetrate the blood‒brain barrier[68], was employed in our previous study and alleviated pain behaviors in pulpitis rats[16]. Thus, injected BBG i.p. to verify the interplay between P2X7R and exosomes. The western blot, qRT‒PCR, and double immunofluorescence staining results indicated that BBG decreased Rab27a and IL-1β expression, suggesting that microglial P2X7R-mediated exosome secretion may be a novel mechanism of pulpitis pain.

In addition to animal experiments, the release of exosomes from BV2 cells upon P2X7R activation has been associated with activation of the metabotropic glutamate receptor[69]. Similarly, microglia-derived microparticles isolated from traumatic brain injury models led to an increase in P2X7R expression, which was accompanied by the upregulation of proinflammatory cytokines[70]. Exosomes from HMC3 cells have been implicated in various conditions, including Alzheimer's disease, brain injury, and neurodegenerative diseases[71–75]. LPS, a strong proinflammatory agent, has been widely applied in in vitro models of inflammation[75, 76]. To confirm the role of P2X7R in exosome secretion, we cultured microglia in vitro and successfully extracted exosomes from them. Through a series of experiments, we found that the levels of microglial P2X7R and exosomes increased, which was accompanied by an increase in IL-1β expression after LPS stimulation. Moreover, IL-1β and P2X7R were detected in inflammatory exosomes, suggesting that persistent inflammatory responses occur due to increased exosome secretion. Previous reports have indicated that the activation of P2X7R induces IL-1β efflux from microglia-derived EVs into target cells[19]. Hence, it is highly conceivable that the excitability of neurons and the production of pain are closely related to the inflammatory signals packaged into microglial exosomes. Notably, we found that inhibiting either P2X7R or exosome secretion produced similar anti-inflammatory effects. In other words, the exosome inhibitor GW4869 and the P2X7R antagonist oxATP reduced LPS-induced microglial inflammatory responses and the degree of exosome secretion, providing evidence for the involvement of P2X7R-mediated exosome secretion in inflammation. The activation of P2X receptors and the subsequent increase in Ca^2+^ influx may be responsible for the increased exosome secretion observed[77]. Moreover, inhibiting Rab27a expression did not affect the morphology of the exosomes but rather reduced the volumes of exosomes in the cell culture supernatants[31]. Our results also revealed that GW4869 and oxATP did not change the diameter of the exosomes but decreased their secretion. Although these results provide some insight into P2X7R-mediated exosome secretion in pain and inflammation, there are several limitations in this study. Compared with the in vivo pulpitis rat model, the pain behaviors could not be observed in the LPS-treated microglia culture. Besides, rat pulpitis is more likely the results of comprehensive factors, including gram-negative bacteria, gram-positive bacteria, pulp tissues, trigeminal ganglion and brain et al., but using LPS to mimic pulpitis-induced microglial inflammation may be the result of a singular factor, which acknowledged certain difficulties to fully mimic pulpitis model[78, 79]. Moreover, future investigations employing proteomic, lipidomic, and transcriptomic analyses of exosomes will elucidate their composition, potentially revealing the underlying mechanisms governing their secretion and role in the inflammatory process.

Overall, our study revealed that Rab27a was upregulated in the MDH of rats with pulpitis and that the exosomes were enriched in IL-1β, indicating increased exosome secretion during the occurrence of pain. Additionally, both the exosome inhibitor and P2X7R antagonist suppressed exosome secretion, which led to pain relief. These findings suggest a potential role for microglial exosomes in the P2X7R-mediated inflammatory response in pulpitis-induced pain.

## Conclusion

**Fig. 8:**
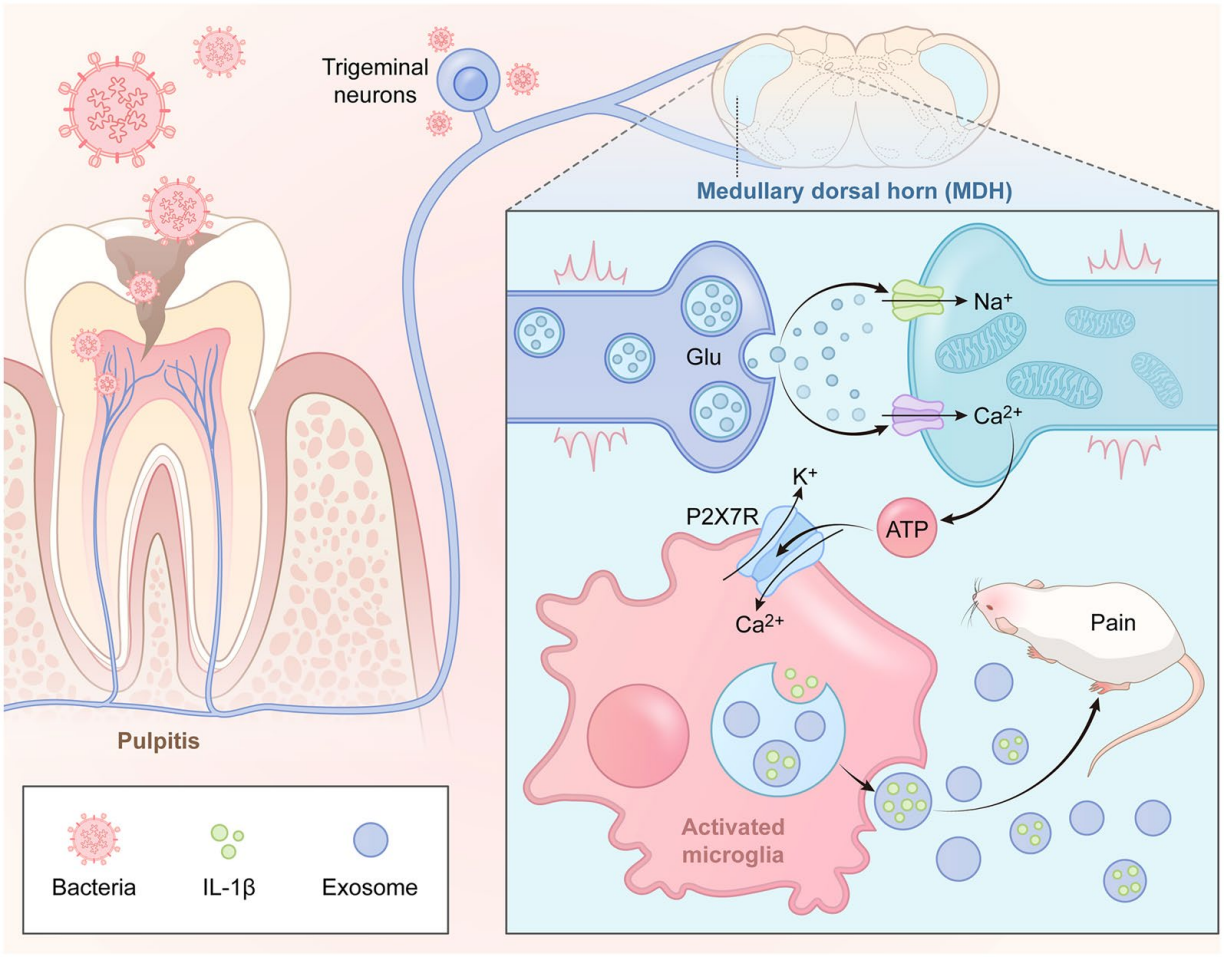
Schematic diagram of the mechanism of pulpitis-induced pain. Following tooth destruction, inflammatory cells infiltrate and disseminate within the dental pulp, resulting in the generation of neural impulses. Activation of microglial P2X7R triggers the secretion of inflammatory exosomes carrying detrimental signals, including IL-1β. Ultimately, the inflammatory mediators exert their effects on neurons, inducing pulpitis pain

In summary, our study suggests that exosomes could serve as novel potential biomarkers for pulpitis-induced pain. Following the onset of pulpitis, the increased secretion of the IL-1β from exosomes is crucial for sustaining persistent pain, and this mechanism is regulated by microglial P2X7R in the MDH (Fig. 8). This investigation highlights the pivotal role of microglial P2X7R in mediating exosome secretion, emphasizing that inhibiting P2X7R could be a therapeutic strategy for alleviating pulpitis-induced pain.

## Supplementary Information

Additional file 1.

Additional file 2.

Additional file 3.

## Data Availability

All data generated or analyzed during this study are included in this article.

## Abbreviations

MDH: Medullary dorsal horn
P2X7R: P2X7 receptor
BBG: Brilliant Blue G
LPS: Lipopolysaccharide
OXATP: Oxidized ATP
MEM: Minimum Eagle's medium
PBS: Phosphate buffered saline
DMSO: Dimethyl sulfoxide
QRT-PCR: Quantitative real-time PCR
CNS: Central nervous system
TEM: Transmission electron microscopy
CCI: Chronic compression injury
NSMase: Neutral sphingomyelinase
