# Supplementary material for: Enhanced exosome secretion regulated by microglial P2X7R in the medullary dorsal horn contributes to pulpitis-induced pain
Source: Cell Biosci. 2025 Feb 22;15:28. doi: 10.1186/s13578-025-01363-4 (PMC11847359; doi:10.1186/s13578-025-01363-4)

Supplementary Figures. Original western blot images

Source_DATA_Figure.2c

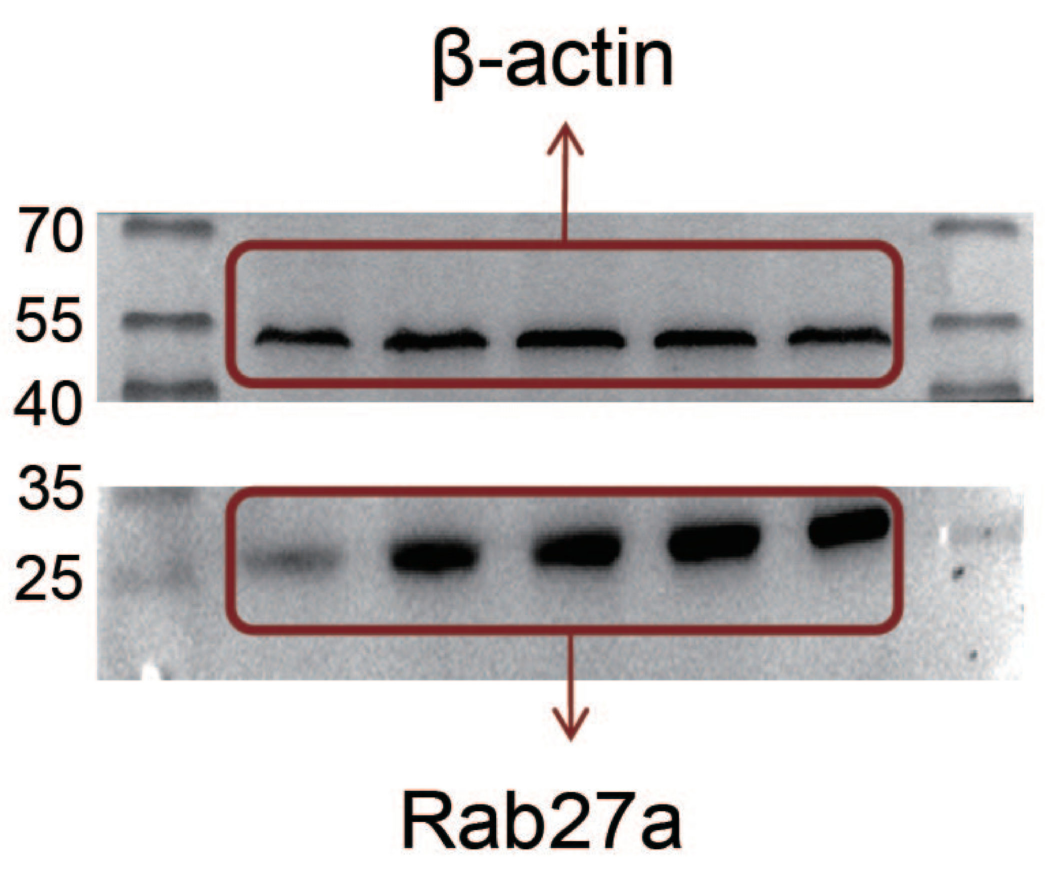

Source_DATA_Figure.3c

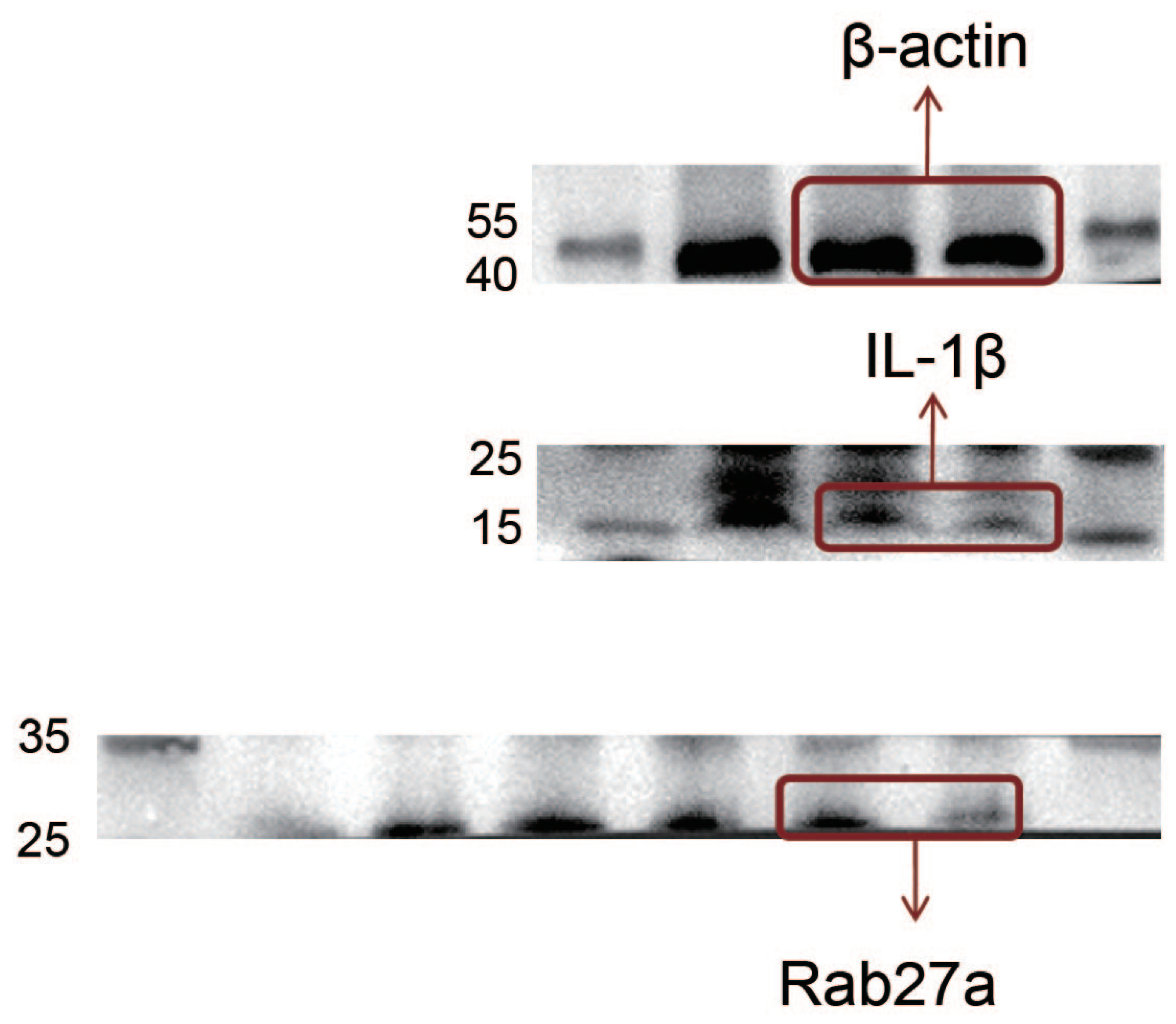

Source_DATA_Figure.4d

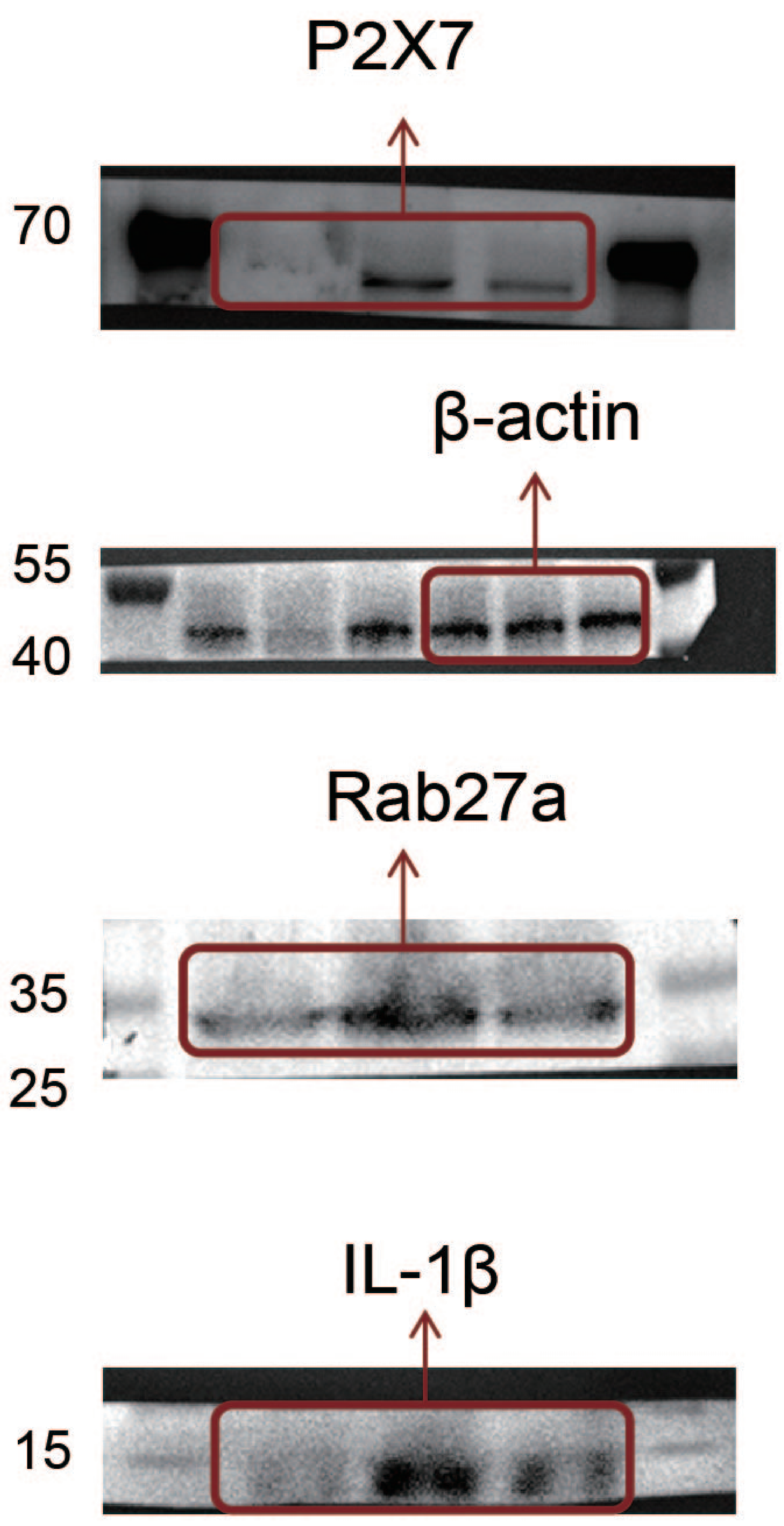

Source_DATA_Figure.5c

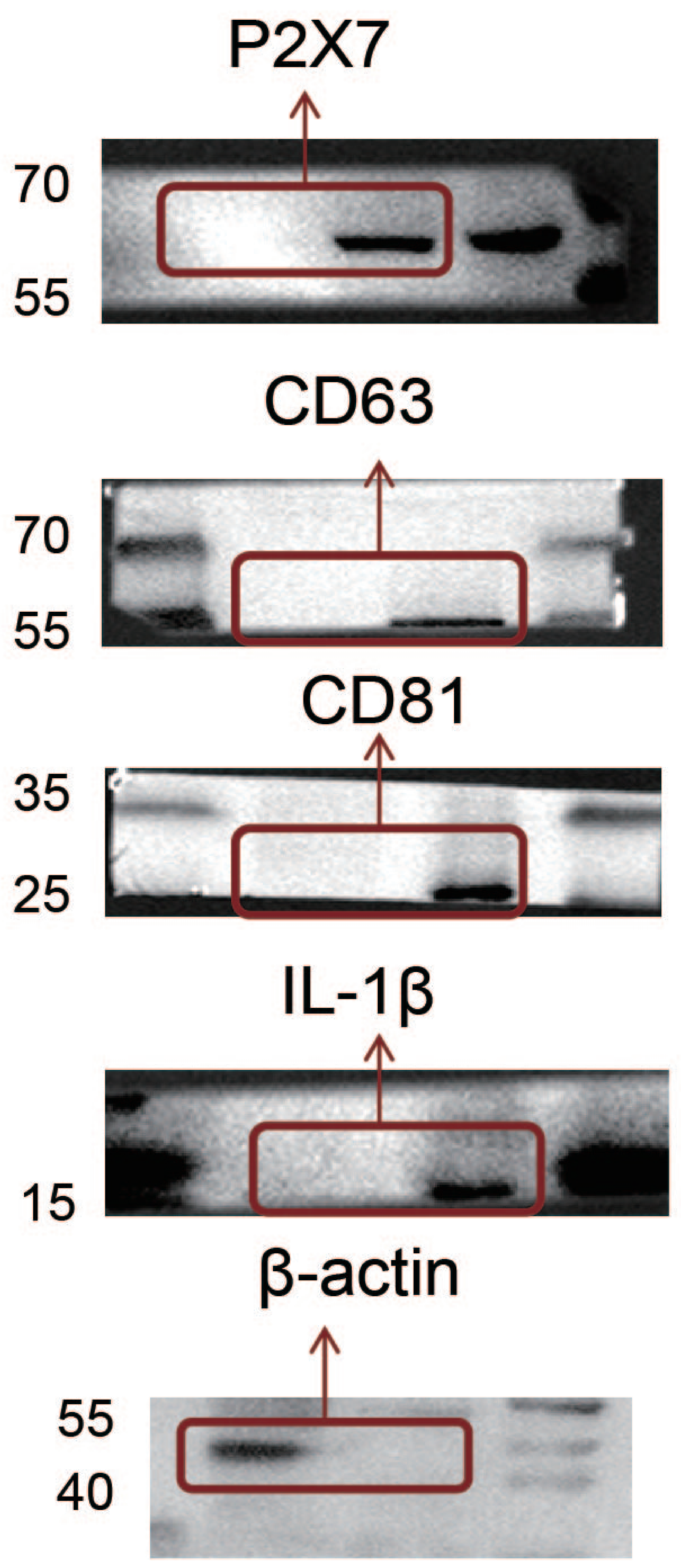

Source_DATA_Figure.6c

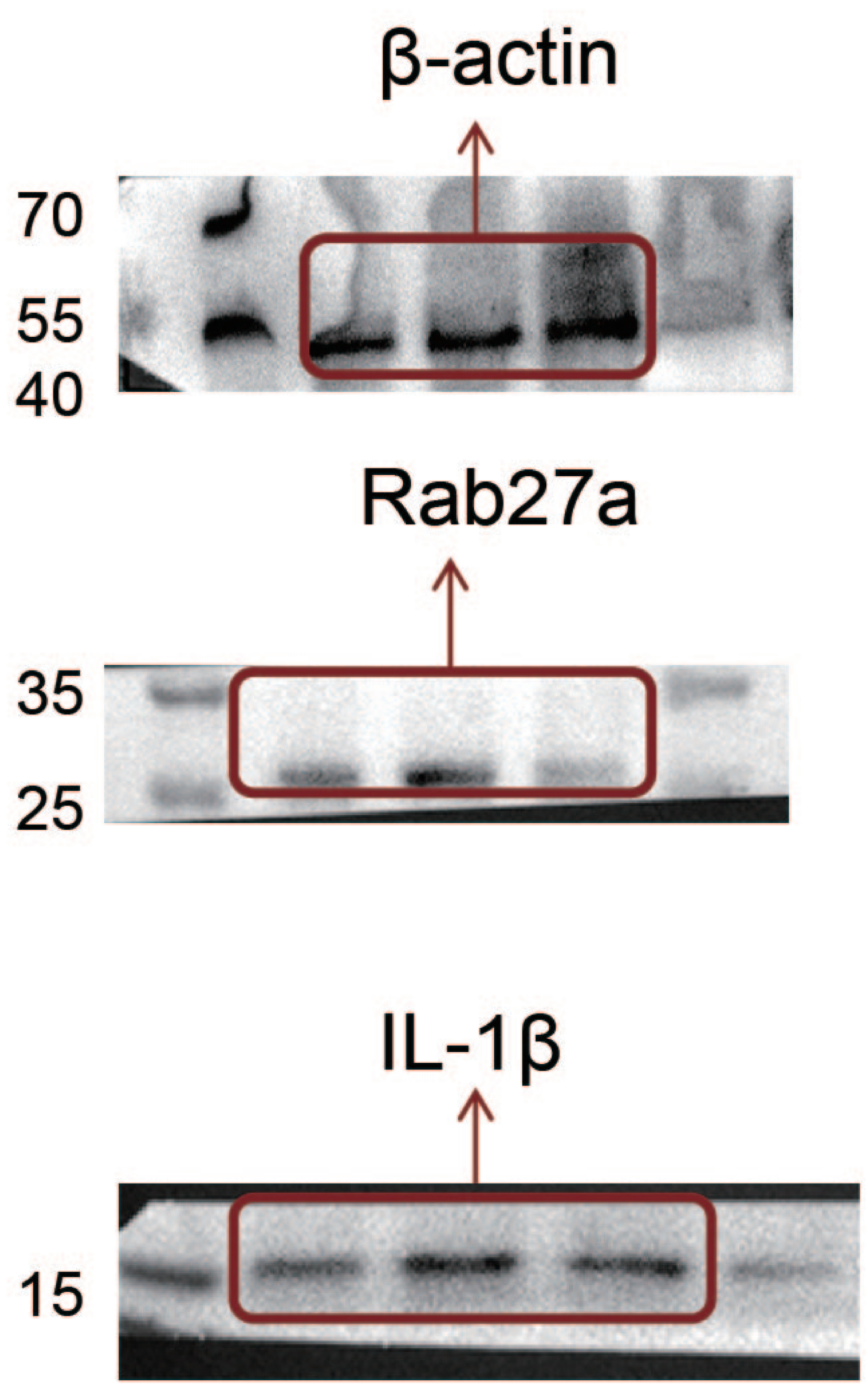

Source_DATA_Figure.7a

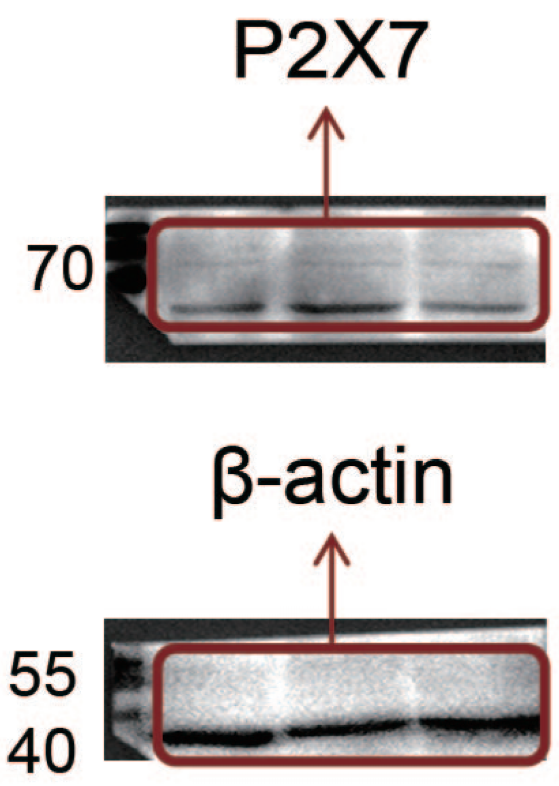

Supplement: Supplementary file 1 — Additional file 1. [file 13578_2025_1363_MOESM1_ESM.pdf]
