# Supplementary material for: Enhanced exosome secretion regulated by microglial P2X7R in the medullary dorsal horn contributes to pulpitis-induced pain
Source: Cell Biosci. 2025 Feb 22;15:28. doi: 10.1186/s13578-025-01363-4 (PMC11847359; doi:10.1186/s13578-025-01363-4)
Supplement: Supplementary file 3 — Additional file 3. [file 13578_2025_1363_MOESM3_ESM.docx]

**Supplementary Table 1.** **Antibodies for Immunofluorescence staining**

| **Antibody** | **Identifier** | **Brand** | **Proportion** |
| --- | --- | --- | --- |
| Mouse anti-P2X7R | sc-514962 | Santa Cruz | 1:100 |
| Goat anti-Iba-1 | Ab5076 | Abcam | 1:400 |
| Rabbit anti-Rab27a | 69295S | Cell | 1:200 |
| Donkey-anti mouse IgG Alexa Fluor 594 | ab150108 | Abcam | 1:1 000 |
| Donkey-anti goat IgG Alexa Fluor 594 | ab150132 | Abcam | 1:1 000 |
| Goat-anti rabbit IgG Alexa Fluor 594 | A23420 | Abbkine | 1:1 000 |
| Donkey-anti rabbit IgG Alexa Fluor 488 | ab150073 | Abcam | 1:1 000 |

**Supplementary Table 2. Antibodies for Western blot**

| **Antibody** | **Identifier** | **Brand** | **Proportion** |
| --- | --- | --- | --- |
| Mouse anti-P2X7R | sc-514962 | Santa Cruz | 1:500 |
| Rabbit anti-IL-1β | ab254360 | Abcam | 1:1 000 |
| Rabbit anti-Rab27a | 69295S | Cell Signaling Technology | 1:1 000 |
| mouse anti-β-actin | sc-47778 | Santa Cruz | 1:1 000 |
| Rabbit anti-CD63 | ab217345 | Abcam | 1:1 000 |
| Rabbit anti-CD81 | ab109201 | Abcam | 1:1 000 |
| HRP-conjugated secondary antibody against mouse | GTX300120 | GeneTex | 1:3 000 |
| HRP-conjugated secondary antibody against rabbit | GTX300119 | GeneTex | 1:3 000 |

**Supplementary Table 3. Sequences of the primers for Quantitative real-time PCR**

| **Gene Species** | **Primer type** | **Sequence 5’-3’** |
| --- | --- | --- |
| Rab27a Rat | Forward | GAACGTAAACAGATGGCGGC |
| Rab27a Rat  IL-1β Rat  IL-1β Rat | Reverse  Forward  Reverse | CTGGGCTGAACCACGGATAA  GAACGTAAACAGATGGCGGC  CTGGGCTGAACCACGGATAA |
| β-actin Rat | Forward | CAAGCAGCAGGAATTGGAACG |
| β-actin Rat | Reverse | CTCATCCATTGACTGCCCCA |
| P2X7 Human | Forward | AGCGGAAAGAGCCTGTCATC |
| P2X7 Human | Reverse | ACGAAGAAAGAGTTCCCCTGC |
| Rab27a Human | Forward | TTGTGGTATTTCCTCAGGTGGC |
| Rab27a Human | Reverse | CTACACCAGAGTCTCCCAAAGC |
| IL-1β Human | Forward | ATGATGGCTTATTACAGTGGCAA |
| IL-1β Human | Reverse | CCTTGCTGTAGTGGTGGTCG |
| GAPDH Human | Forward | AGAAGGCTGGGGCTCATTTG |
| GAPDH Human | Reverse | AGGGGCCATCCACAGTCTTC |
